# Pharmacological Treatment of Medication-Related Osteonecrosis of the Jaw (MRONJ) with Pentoxifylline and Tocopherol

**DOI:** 10.3390/jcm14030974

**Published:** 2025-02-03

**Authors:** Łukasz Słowik, Ewa Totoń, Aleksy Nowak, Aleksandra Wysocka-Słowik, Maciej Okła, Zuzanna Ślebioda

**Affiliations:** 1 Department of Maxillofacial Surgery, Poznan University of Medical Sciences, 61-701 Poznan, Poland; nowak.aleksy@spsk2.pl (A.N.); maciejokla@ump.edu.pl (M.O.); 2 Department of Clinical Chemistry and Molecular Diagnostics, Poznan University of Medical Sciences, 61-701 Poznan, Poland; etoton@ump.edu.pl; 3 Department of Dental Surgery, Periodontology and Oral Mucosa Diseases, Poznan University of Medical Sciences, 61-701 Poznan, Poland; wysocka.slowik@ump.edu.pl

**Keywords:** medication-related osteonecrosis of the jaws, bisphosphonates, pentoxifylline, tocopherol

## Abstract

**Background:** This study aimed to evaluate the efficacy of pentoxifylline and tocopherol therapy in patients with medication-related osteonecrosis of the jaw (MRONJ). **Methods:** During this study, 43 patients participated, including 21 women and 22 men with a mean age of 66.8 years, who showed 63 areas of osteitis altogether. The diagnosis was made based on X-ray imaging and histopathological findings. All the subjects received pharmacological treatment with pentoxifylline 400 mg and tocopherol 400 IU. The study scheme consisted of initial observation and two follow-up examinations every 5–6 months. MRONJ severity, peripheral blood parameters, and CRP levels were evaluated. The obtained results were statistically analyzed. **Results**: Complete remission occurred in 46% of the subjects, with a higher rate among those taking bisphosphonates intravenously compared to oral administration. The efficacy of pentoxifylline and tocopherol treatment was not influenced by gender or lesion location. Moreover, the worst response to treatment was observed in the group with the highest disease stage, as determined in the initial study. **Conclusions:** Pentoxifylline and tocopherol therapy in MRONJ was effective in patients taking oral and intravenous bisphosphonates, in patients with osteoporosis, and undergoing oncological treatment. This treatment approach allows surgery to be avoided or significantly reduced. The good response to pharmacotherapy observed in patients with early stages of MRONJ shows an urgent need to monitor the patients treated with bisphosphonates carefully to diagnose MRONJ at the initial phase.

## 1. Introduction

The first case reports of bisphosphonate-related osteonecrosis of the jaw (BRONJ) appeared in the literature in the early 2000s, and osteonecrosis was due to zoledronic acid and pamidronic acid [1]. By 2014, it was inevitable that the group of medications causing jawbone necrosis was broader. As a result, the American Association of Oral and Maxillofacial Surgeons (AAOMSs) proposed changing the name of the entity to medication-related osteonecrosis of the jaw (MRONJ) [2]. It has been challenging for dentists and their patients for over 20 years. As more and more cases emerged, AAOMS established a definition of this type of inflammatory lesion that remains unchanged to this day. Three elements are required to diagnose MRONJ: the absence of radiotherapy and metastatic lesions in the craniofacial region, a history of antiresorptive or antiangiogenic drug administration, and an intra- or extraoral fistula that probes to necrotic bone [2,3].

Osteoclasts are the cells responsible for bone degradation, and they play a crucial role in the physiological remodeling of bone tissue [4,5]. Bisphosphonates, synthetic pyrophosphate derivatives with an antiresorptive effect, accumulate in osteoclasts leading to their death. These drugs are used in the treatment of bone cancer, cancer metastases to bone, Paget’s disease, multiple myeloma, osteoporosis, congenital bone fragility, hypercalcemia, and other conditions in which increased bone fragility is present [3,4,5].

Complications of bisphosphonate therapy are rarely noted. Intravenous administration of bisphosphonates can increase levels of inflammatory cytokines, manifesting as fever, fatigue, nausea, and muscle pain. Less commonly, hypocalcemia, kidney damage, and orbital inflammation are diagnosed. Another potential complication, which is jawbone necrosis, may arise from oral infections, smoking, and poor oral hygiene. Awareness of bisphosphonates’ adverse effects among healthcare professionals and patients is a crucial issue in MRONJ prevention [6,7,8].

The most common location of MRONJ is the mandible, and complications of oncological intravenous therapy are more likely to cause necrosis than the treatment of osteoporosis with oral drugs [9].

Drug-induced necrosis of the jawbones mainly affects the elderly population [10]. Surgical treatment of MRONJ carries the risks associated with surgery itself and with general anesthesia. Therefore, pharmacological methods are researched to offset the potential risks associated with surgical therapy. Among non-invasive methods, neodymium-Yag laser, hyperbaric oxygen, teriparatide, pentoxifylline, and tocopherol have been studied [11]. The PENTO (PENtoxifylline—TOcopherol) regimen is a non-invasive pharmacological method that may potentially treat and prevent MRONJ [12]. Pentoxifylline (PTX) is considered a safe drug that increases blood flow by reducing its viscosity. The mechanism of action of pentoxifylline includes making erythrocytes more flexible, which facilitates their passage through microcirculation and improves tissue perfusion [13,14]. In nature, eight substances have vitamin E activity: alpha-, beta-, gamma-, and delta-tocopherols; and alpha-, beta-, gamma-, and delta-tocotrienols. The best known of these, alpha-tocopherol, is characterized by strong antioxidant properties. Beneficial preventive effects of vitamin E have been found in neurodegenerative disorders, Alzheimer’s disease, breast cancer chemoprevention, cardiovascular diseases, and atherosclerosis [14,15,16].

Due to the disadvantages of the surgical treatment approach, there has been a growing interest in non-invasive treatment strategies for MRONJ. Thus far, reports of MRONJ treatment with PENTO have been scarce, and there are currently no well-defined treatment guidelines for MRONJ’s pharmacologic management. Benefits related to symptom reduction and remission of clinical lesions have been presented by Cavalcante et al., Kemp et al., and de Morais et al. [17,18,19]. This treatment approach allows surgery to be avoided or significantly reduced. Available publications indicate the need for further studies to determine the optimal management protocol for patients with drug-induced osteitis of the mandible. Observations of the therapy’s efficacy over a longer time are needed. They may help to create an algorithm to indicate the patients for whom the therapy will be the most beneficial.

This study aimed to evaluate the efficacy of pentoxifylline and tocopherol therapy in patients with MRONJ, taking into account the route of bisphosphonate administration (intravenous; *iv* or oral; per os; *po*), the patient’s gender, the location of the lesions, and the diagnosis of the underlying disease for which antiresorptive drugs were applied.

## 2. Materials and Methods

The study group consisted of 43 subjects, including 21 women and 22 men with MRONJ, who were diagnosed with 63 foci of osteitis. The mean age of the subjects was 66.8 years. They were treated at the Outpatient Clinic of the University Clinical Hospital in Poznań. They were referred by their family doctors or dentists. The diagnosis was based on the clinical picture, radiological findings, and histopathological analysis. All the patients diagnosed with MRONJ in the period of this study who agreed to participate in this project were enrolled. Comorbidities or habits were not the excluding criteria.

**Figure 1:**
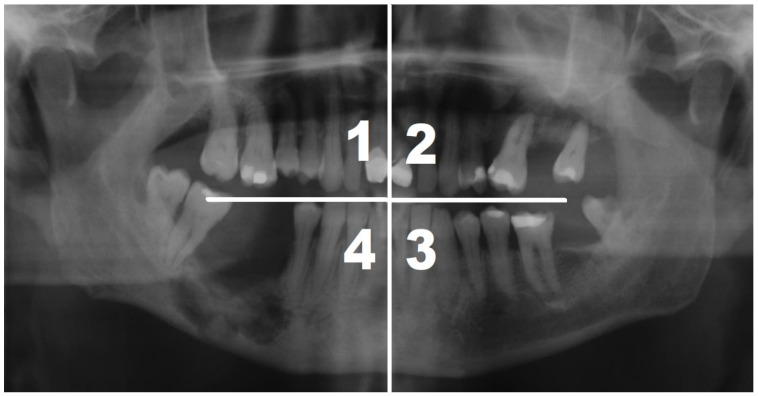
This study used oral cavity division into quadrants (1–4) to describe the location of lesions.

The observation scheme included an initial and two follow-up examinations performed at 5–6 month intervals. During the initial examination, gender, age, MRONJ severity, duration of MRONJ, and location of lesions were determined. The underlying disease, an indication to apply antiresorptive drugs, was identified, and history regarding systemic comorbidities and treatment was collected. Cell Blood Count (CBC) was performed in all participants, and C-reactive protein (CRP) levels were determined. The oral cavity was divided into four quadrants for data analysis: 1—right maxillary, 2—left maxillary, 3—left mandibular, and 4—right mandibular (Figure 1).

Pharmacological treatment was implemented in all eligible patients. The therapy included pentoxifylline 400 mg (2 × 1 400 mg tablet/24 h) and tocopherol 400 IU (2 × 1 400 IU tablet/24 h). The treatment was administered continuously throughout this study. Drug dosages were determined based on literature reports by other researchers. An equivalent regimen was presented in the study by Owosho et al., Seo et al., and Epstein et al.; a comparable one was by Dissard et al. and Dalamian et al. Here, pentoxifylline was administered at 400 mg/day combined with tocopherol at the dose of 500 IU/day. The pentoxifylline dosage was designed to avoid severe adverse cardiovascular effects; the tocopherol dosage was determined to supply sufficient antioxidant activity [13,20,21,22,23,24].

During treatment, patients underwent a follow-up examination twice, at 5–6 month intervals. It included an assessment of MRONJ severity, drug tolerance, basic peripheral blood parameters (CBC), and CRP. The severity of MRONJ was assessed in clinical examination and radiological examination according to the 4-stage AAOMS classification. The classification scheme is shown in Table 1. Complete remission was defined as progression to Stage 0 at the third examination, and improvement was defined as a reduction in staging by one or more grades but not to Stage 0.

This study was approved by the Poznan University of Medical Sciences Ethics Committee (approval code: 284/17) and complied with the Declaration of Helsinki’s guidelines. All the patients were informed in detail about the nature of this study before consent was obtained for participation in this project.

Data were organized in MSExcell^®^ spreadsheets and presented descriptively. The chi-square test, the difference test between two proportions, and the logistic regression model were used where appropriate, with *p*-values lower than 0.05 considered significant. Dell Statistica (data analysis software system), version 13 (Dell Inc., 2016; Palo Alto, CA, USA), was used for the purpose of this analysis.

## 3. Results

Table 2 depicts the demographic characteristics of the study group, with the route of bisphosphonate application, MRONJ location, and the number of affected oral cavity quadrants.

There was almost equal sex distribution in the study group (21 females and 22 males). Most patients were between 60 and 69 years old (44.2%). Intravenous application of antiresorptive therapy was introduced in 33 patients (76.7%) in whom 50 MRONJ lesions where detected (79.4%). The remaining 10 patients (23.3%), who altogether developed 33 pathologic lesions (20.6%), received oral treatment. All the subjects treated intravenously had been diagnosed with cancer, while the group treated orally received bisphosphonates for osteoporosis. Most MRONJ lesions were located in the mandible (47 locations; 74.6%), mainly in the fourth quadrant. Pathologic lesions were usually situated simultaneously in one or two quadrants (26 and 28 locations, respectively). In no patients were all four quadrants affected with pathologic lesions simultaneously.

Table 3 shows MRONJ staging concerning the lesion’s location and the route of antiresorptive treatment administration on three subsequent examinations.

The correlation between MRONJ staging in the preliminary and final examinations in the study group is presented in Table 4.

Complete remission in the final study [Stage 0] was achieved in 11% of the lesions (seven locations) whose stage at the initial study was defined as Stage I. Complete remission was also found in 29% of Stage II lesions (18 locations) in the first study and 6% of Stage III lesions (4 locations) in the first study. The same lesion stage after therapy compared to the initial stage was presented by 16% of Stage III lesions, 19% of Stage II lesions, and 2% of Stage I lesions. There was a statistically significant dependence between the results of the first and third examinations (Table 4). 

Table 5 depicts the correlation between MRONJ staging in preliminary and final examinations depending on the route of antiresorptive drug administration.

In the group that received intravenous bisphosphonate treatment, remission after PENTO therapy was achieved in 14% of cases (7 locations) of Stage I MRONJ, in 28% (14 locations) of Stage II MRONJ, and in 6% (3 locations) of Stage III MRONJ found in the initial examination. In 4% of cases (2 locations), there was a deterioration from Stage II to Stage III on the examination after 11–12 months of PENTO therapy.

Comparing the results of the final examination with the initial examination in the group taking oral bisphosphonates showed that remission occurred in 31% of Stage II cases and 7.5% of Stage III cases. Results distribution and analysis show no correlation between examination III and I in patients taking oral bisphosphonates.

Table 6 shows the treatment efficacy by comparing the patient’s condition on the initial and the last examination, performed after 11–12 months of PENTO therapy.

Pharmacological therapy brought about complete remission of the lesions in 29 cases, improvement in 9 cases, no change in the grade of the lesions in 23 cases, and progression of the lesion in 2 cases. The dependence between the results obtained in the first and third examinations was statistically significant.

Table 7 depicts the treatment efficacy estimated based on comparing the patient’s condition on the initial and the last examination, performed after 11–12 months of PENTO therapy, regarding the route of antiresorptive drug administration, gender, and the lesions’ location. The chi-square test was used for the analysis.

At the end of observation, in patients with the *iv* route of bisphosphonate administration, 48% of the lesions were cured completely, 12% of the cases improved, 36% showed no change, and 4% worsened. In contrast, in those with the *po* route of administration, 38.5% of the lesions studied were cured entirely, 23% of the cases improved, and the condition of 38.5% of the lesions did not change (Table 7).

The dependence between treatment efficacy and an underlying disease is identical to the dependence between treatment efficacy and the drug administration route because the administration route was *iv* for all cancer patients and *po* for all osteoporosis patients.

Complete remission occurred in 40.5% of the women and half of the men. Improvement was observed in 15% of the women and 14% of the men. No change was observed in 40.5% of the women and 33% of the men. Deterioration occurred in one case in both sexes (Table 7).

Table 8 illustrates the treatment efficacy estimated by comparing the patient’s condition on the initial and the last examination, performed after 11–12 months of PENTO therapy, regarding the lesions’ location.

Complete remission occurred in 68.5% of the lesions located in the maxilla and in 38% of the lesions in the mandible. Improvement was noted in 13% of the maxillary and 15% of the mandibular lesions. Furthermore, 18.5% of the maxillary and 43% of the mandibular lesions remained unchanged (Table 8).

## 4. Discussion

We present the therapy of 43 MRONJ patients according to the PENTO scheme. All subjects were treated with pentoxifylline 400 mg (2 × 1 tablet/24 h) and tocopherol 400 IU (2 × 1 tablet/24 h). The therapeutic algorithm was established based on literature reports from other researchers. An equivalent regimen was presented in the study by Owosho et al. [20], Seo et al. [13], and a comparable one by Dissard et al. and Delanian et al. [23,24]. Here, pentoxifylline was administered at 400 mg/day combined with tocopherol at the dose of 500 IU/day. The drug dosages were adjusted to avoid severe cardiovascular effects and achieve sufficient antioxidant activity. There were no serious side effects during the study period. While the effect of PTX may be seen within 2 to 4 weeks, it is recommended that treatment be continued for at least 8 weeks [13]. Higher doses of both drugs were used in the Dos Anjos et al. study [25], where doses of 1200 mg/day pentoxifylline and 1200 IU/day tocopherol were applied.

In the present analysis, we attempted to evaluate the efficacy of pharmacological treatment in groups of patients, considering gender, location of lesions, route of bisphosphonate administration, and the diagnosis of the underlying disease, which required antiresorptive treatment. This evaluation aimed to identify the patients for whom drug therapy would benefit most.

Our study group had almost equal sex distribution (21 females and 22 males). Most patients were between 60 and 69 years old (44.2%). Intravenous application of antiresorptive therapy, which was required in patients with cancer, was introduced in 33 patients (76.7%) and 50 locations (79.4%). The remaining 10 patients (23.3%) with 33 pathologic lesions (20.6%) received oral bisphosphonates for osteoporosis. According to the systematic review by Cavalcante et al., MRONJ was more common in elderly women. In the studies included in the review, the treatment of osteoporosis involved only the prescription of bisphosphonates, such as alendronate, ibandronate, and zoledronate. They found a stronger association between osteoporosis treatment and MRONJ compared to treatments for other medical conditions [17]. In a comprehensive analysis of 661 cases of MRONJ, Ling-Ying et al. also indicated advanced age as a significant risk factor for the disease [26].

The results of our observations and those of other authors confirm the effectiveness of PENTO therapy in treating MRONJ [18,19,20,21,22,23,24,25]. We also confirmed the efficacy of this treatment approach in the previously published case report, where the resolution of inflammatory symptoms and pain reduction was achieved in two patients with multiple myeloma after introducing the PENTO protocol. It helped avoid mutilating surgical procedures [26]. In the current study, 46% of the patients experienced complete disease remission following treatment. These data are consistent with reports by other investigators, where the percentage of patients cured ranged from 45 to 100% [13,18,19,20,21,22,23,24,25]. Bone healing was observed in all nine patients included in the Seo et al. study, with an average reduction of 74% in bone-exposed areas [13]. A complete treatment course cured 76.5% of the subjects, according to the Dissard et al. report [23], while 74% recovered in the Epstein et al. study [22]. The rate of complete healing was also comparable to the Dos Anjos et al. report, where it reached 76% of the study population [25]. The PENTO protocol relieved painful symptoms in all seven MRONJ patients and resulted in significant new bone formation at the final follow-up in the Owosho et al. study [20]. Recovery in all the study participants (100%) was reported by Magremanne et al. [21].

Moreover, the present study suggests that the worst response to treatment was observed in the group with the highest disease stage, as determined in the initial study (Table 4). That could result from the impaired ability of the drugs to penetrate severely damaged bone. Potentially higher doses of PENTO or a prolonged treatment duration could be beneficial in those patients.

Complete remission was achieved in 11% of cases classified as Stage I and in 6% of lesions classified as Stage III on the preliminary examination. In the 2023 study by Magalhães et al., all patients starting PENTO therapy in MRONJ Stage I achieved complete remission after 30 days of treatment, and one of the patients underwent sequestrotomy. In this study, pharmacotherapy was combined with photodynamic therapy. The medication protocol included pentoxifylline, tocopherol, systemic and topical antibiotic therapy, and analgesics when necessary. Pentoxifylline 400 mg and tocopherol 400 IU were administered every eight hours for 30 days [27]. The daily dose was, therefore, higher than in our therapeutic algorithm, although the treatment period was much shorter. The good response observed in patients with Stages I and II confirms the need for careful attendance of the patients treated with bisphosphonates to diagnose MRONJ as soon as possible and implement PENTO treatment at an early stage of the disease progression [27]. In the randomized study by Calapinto et al., PENTO was applied as an adjuvant therapy to the surgical management of MRONJ Stage I to affect the disease’s progress by improving blood circulation and making the surgery as minimally invasive as possible [28]. Applying the PENTO protocol before surgery aimed to counteract the chronic ischemic condition and overcome the oxidative stress associated with the rebound effect by improving blood circulation. The pre-operative pharmacological preparation was used to reduce the risk of osteonecrosis. According to the authors, pentoxifylline prevented chronic ischemia, while tocopherol reduced the toxicity of oxygen-free radicals, restoring adequate blood flow to the bone [28]. It should be emphasized that reactive oxygen species (ROS) may arise in both ischemia and reperfusion periods. Limited oxygen availability during the ischemic period is associated with acidosis, energy depletion, and alterations of ion homeostasis, leading to cell death. Oxygen free radicals (OFR) are produced immediately following reperfusion due to the sudden reintroduction of high oxygen tensions. That may lead to oxidative damage of cell structures. The mechanism of oxidative stress after reperfusion and how it affects injuries is multifactorial [29]. Data from the Cuddihy et al. study confirmed that at higher levels of α-tocopherol, mitochondrial ROS production is decreased. This finding might be relevant to oxidative stress in specific tissues, which could lead to localized depletion of α-tocopherol or inadequate dietary availability of α-tocopherol in vulnerable populations (e.g., surgical patients and smokers) [30].

Our study found no statistically significant relationship between the effectiveness of treatment with the PENTO regimen and the route of application of antiresorptive drugs. We found a more evident clinical improvement in patients on intravenous bisphosphonates than those on oral bisphosphonates (cure in 48% and 38.5% of cases, respectively). The observed trend may be partly due to a significant disproportion in the number of patients who received intravenous vs. oral bisphosphonates in our study, so further analysis of a larger patient population is undoubtedly required. Moreover, all the patients treated intravenously received antiresorptive drugs in the course of cancer, which was their primary diagnosis, while those treated with oral bisphosphonates were diagnosed with osteoporosis.

We also found no statistically significant difference between the effectiveness of PENTO and the gender of the subjects. We noted that MRONJ was more common in the mandible (47 cases) than in the maxilla (16 cases). However, no statistically significant difference existed between the treatment efficacy and lesion location (*p* = 0.1728).

Guidelines for the clinical management of MRONJ remain unclear [2,28]. In Cavalcante et al. and Beth-Tasdogan et al.’s reviews, it was noted that various therapeutic strategies have been used for MRONJ management. They included surgical approaches (conservative, extensive with or without fluorescent light, laser) and non-invasive options, like chlorhexidine 0.12% mouth rinse, antibiotic therapy, hyperbaric oxygen (HBO), low-level laser therapy, plasma-rich protein (PRP), teriparatide, and the PENTO protocol [17,31,32]. A late complication of untreated MRONJ is a tendency to develop pathological fractures. Surgical treatment of the elderly population, stable osteosynthesis, external fixation, or resection is significantly burdensome and associated with long-term rehabilitation [33,34]. Due to the apparent disadvantages of surgical treatment modalities, there has been a growing interest in non-invasive treatment strategies for MRONJ.

Although there is currently no well-defined treatment algorithm for the pharmacologic management of MRONJ, our observations confirm that the PENTO protocol applied in our study group, composed of 400 mg pentoxifylline (2 × 1 tablet/24 h) and 400 IU tocopherol (2 × 1 tablet/24 h), is an effective and safe option. As emphasized in de Morais et al.’s overview, pentoxifylline and tocopherol treatment alone is possible depending on the severity of osteonecrosis and the host’s response. However, careful monitoring of pharmacologically treated MRONJ patients is required to assess the need for concomitant surgery [19].

Other potential non-invasive modalities for MRONJ treatment include hyperbaric oxygen therapy (HBO), which was shown to reduce improvement time and was associated with a higher rate of clinical improvement compared to surgery and antibiotics alone in the two Freiberger et al. studies [35,36]. In the clinical trial, where they compared HBO treatment used in addition to standard care (antiseptic rinses, antibiotics, and surgery) with standard care alone, 17 of 25 HBO-treated patients (68%) improved versus 8 of 21 controls (38.1%). Complete gingival healing occurred in fourteen HBO-treated patients (52%) versus seven controls (33.3%), with no statistically significant differences. Significant differences between HBO and control groups were found for mean improvement time (39.7 vs. 67.9 weeks, respectively) and for pain and quality-of-life scores for physical health [35]. In the case series, 14 of 16 patients (87.5%) improved in stage, and the size and number of lesions decreased after HBO. The patients who continued BP treatment during HBO had a shorter time to failure than those who discontinued the drug [36]. Meanwhile, a recent Cochrane study did not confirm the effectiveness of HBO in the treatment of MRONJ [31]. The authors of this analysis emphasize that after identifying eight randomized clinical trials that evaluated specific methods to improve the healing of MRONJ, namely hyperbaric oxygen (HBO) therapy, fluorescence-guided bone surgery, growth factors such as PRF, concentrated growth factor or bone morphogenic protein 2, and teriparatide, there was insufficient evidence to either claim or refute a benefit of any of these therapies for improved healing of MRONJ [31].

Low-level laser therapy (LLLT) was not shown as an effective treatment option in MRONJ in the Favia et al. retrospective study, which compared surgical and non-surgical treatment approach efficacy. The non-surgical protocol included the use of an antiseptic mouth rinse (chlorhexidine), periodic dental checks, systemic antibiotic administration (ceftriaxone 1 g/i.m. daily and metronidazole 500 mg/per os twice a day for 7 days once a month), monthly low-level laser therapy, consisting of the irradiation of the necrotic bone by diode laser employed with a fiber of 320 μm, a wavelength of 800 ± 10 nm, at the power of 0.5–1 W; and removal of bone sequestra separated from the surface of the exposed bone. All the surgically treated lesions (107) showed complete healing, with the exception of 13.5% of the lesions, all of which were Stage III, which did not completely heal but showed a reduction to Stage I. The 24 non-surgically treated lesions never completely healed and, rather, generally remained stable. Only two cases exhibited a reduction in staging [37].

Teriparatide (TPTD) efficacy was confirmed in Kim et al.’s study [38]. In the TPTD group, 62.5% of the treated subjects showed one stage of improvement, and the other 37.5% demonstrated a marked improvement, including two stages of improvement or complete healing, with not a single case that did not improve. Meanwhile, 60.0% of the non-TPTD group showed one stage of improvement in BRONJ, but 40.0% of the group did not show any improvement in disease status. The clinical improvement of BRONJ was statistically better in the TPTD group after the 6-month treatment (*p* < 0.05). A randomized trial of teriparatide (20 μg/d) or placebo in 57 MRONJ lesions in 37 patients was performed by Sim et al. The results from this study noted an improved rate of resolution of MRONJ, with 45.4% of lesions in patients on teriparatide resolved versus 33.3% in those receiving placebo (OR 0.15 vs. 0.40; *p* = 0.013) and reduced bony defects upon resolution (OR 0.81; *p* = 0.017) [39]. A study of teriparatide treatment of MRONJ by Kwon et al. showed healing in all six patients within 2 months of the therapy [40]. Teriparatide with bone morphogenetic protein (BMP)-2 was evaluated in 17 patients in the Jung et al.’s study. They noted increased bone formation reported with combination therapy than with BMP alone and compared to controls [41].

The overall success of photobiomodulation (PBM) with complete healing was reported as 96% in a report of 241 patients exposed to antiresorptive or antiangiogenic therapy and patients with bone exposure [42]. In this study, PBM was combined with antibiotic therapy and with or without surgical procedures. Meanwhile, a 2021 study by Varoni et al. showed an 88.5% success rate in curing MRONJ after the use of antibiotics and antiseptics [43].

An interesting comparison of the efficacy of several surgical and non-invasive treatment approaches in MRONJ was presented in Fliefel et al.’s systematic review. The highest rate of complete healing was reported for the major surgery (82.1%), followed by the growth factors and teriparatide (81.5%). Ozone therapy efficacy reached 57.8%, guided debridement reached 48%, laser therapy reached 45.3%, and medical treatment reached 45.1%. HBO showed a 26.7% efficacy. A total number of 1571 patients were included, with a mean efficacy in reaching complete remission estimated as 45.2% [44]. That corresponds to our study results, where the rate of complete remission reached 46%.

Future research performed on large study samples in a prolonged manner should, therefore, focus on exploring different dosages of the PENTO approach concerning its potential side effects on the cardiovascular system and evaluating its efficacy in combination with other therapies like the above-mentioned low-level laser therapy and HBO.

This study has some limitations, including a relatively small sample size and the lack of a control group. The study group was not homogenous in terms of comorbidities and habits. The observation period was limited to one year, comparable to the schemes presented by other authors [13,20,21,22], although prolonged follow-up could be beneficial.

## 5. Conclusions

PENTO therapy is effective in treating MRONJ in patients taking oral and intravenous bisphosphonates, treated for osteoporosis, and undergoing oncological treatment. The efficacy of treatment with PENTO was not influenced by the patient’s gender or lesion location. Moreover, the worst response to treatment was observed in the group with the highest disease stage, as determined in the initial study.

This treatment approach allows surgery to be avoided or significantly reduced. The good response to pharmacotherapy observed in patients in the early stages of MRONJ shows an urgent need to carefully monitor the patients treated with bisphosphonates to diagnose MRONJ in the initial phase and implement the PENTO treatment as soon as possible.

Further studies on the mechanism of the PENTO protocol in MRONJ are needed to develop definite recommendations on the dosage and duration of treatment.

## Figures and Tables

**Table 1:** Definition and staging of medication-related osteonecrosis of the jaw (MRONJ) according to the American Association of Oral and Maxillofacial Surgeons [2].

MRONJ Stage	Description
At risk	No apparent necrotic bone in patients who were treated with oral or intravenous bone-modifying agents
0	No clinical evidence of necrotic bone but with nonspecific symptoms or clinical and radiographic findings
1	Exposed and necrotic bone or fistulas that probe to bone in asymptomatic patients with no evidence of infection
2	Exposed and necrotic bone or fistulas that probe to bone associated with infection as evidenced by pain and erythema in the region of exposed bone with or without purulent drainage
3	Exposed and necrotic bone or fistula that probes to the bone in patients with pain, infection, and one or more of the following: exposed and necrotic bone extending beyond the region of alveolar bone (i.e., inferior border and ramus in mandible, maxillary sinus, and zygoma in maxilla) resulting in pathologic fracture, extraoral fistula, oral antral or oral nasal communication, or osteolysis extending to the inferior border of the mandible or sinus floor

**Table 2:** Demographic characteristics of the study group, including the route of bisphosphonate application, MRONJ location, and the number of affected oral cavity quadrants.

Patient Characteristics			Patients (Total: 43 Cases)	MRONJ Location (Total: 63 Locations)
Sex	Female		21	27
	Male		22	36
Age at diagnosis	50–59		9	12
	60–69		19	28
	70–79		10	15
	80–89		5	8
Antiresorptive therapy	po		10	13
	iv		33	50
MRONJ location	Maxilla quadrant	1st	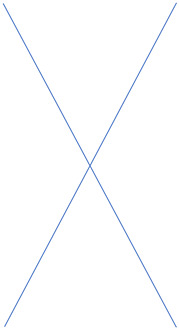	7
		2nd		9
	Maxilla total			16
	Mandible quadrant	3rd		19
		4th		28
	Mandible total			47
Number of occupied quadrants	One	po		7
		iv		19
	Two	po		6
		iv		22
	Three	po		0
		iv		9
	Four	po		0
		iv		0

**Table 3:** MRONJ staging concerning the lesions’ location and the route of antiresorptive drug administration on subsequent examinations.

MRONJ Location (Total 63 Locations)			Examination I (Preliminary)	Examination II after 5–6 Months of PENTO Therapy	Examination III after 11–12 Months of PENTO Therapy
MRONJ stage:		Location			
	Stage 0	Maxilla	0	3	11
		Mandible	0	9	18
		Total	0	12	29
	Stage 1	Maxilla	5	7	1
		Mandible	3	9	5
		Total	8	16	6
	Stage 2	Maxilla	9	4	4
		Mandible	26	18	12
		Total	35	22	16
	Stage 3	Maxilla	2	2	1
		Mandible	18	11	11
		Total	20	13	12
MRONJ stage:		Antiresorptive therapy			
	Stage 0	*po*	0	2	5
		*iv*	0	10	24
		Total	0	12	29
	Stage 1	*po*	0	3	2
		*iv*	8	13	4
		Total	8	16	6
	Stage 2	*po*	8	6	4
		*iv*	27	16	12
		Total	35	22	16
	Stage 3	*po*	5	2	2
		*iv*	15	11	10
		Total	20	13	12

**Table 4:** Correlation between MRONJ staging in the preliminary and final examination.

		Examination III After 11–12 Months of PENTO Therapy				*p*
		Stage 0	Stage I	Stage II	Stage III	
Examination I (Preliminary)	Stage I	7 (11%)	1 (2%)	0 (0%)	0 (0%)	0.0004
	Stage II	18 (29%)	3 (5%)	12 (19%)	2 (3%)	
	Stage III	4 (6%)	2 (3%)	4 (6%)	10 (16%)	

**Table 5:** Correlation between MRONJ staging in preliminary and final examination depending on the route of antiresorptive drug administration.

*iv* (N = 50)		Examination III After 11–12 Months of PENTO Therapy				*p*
		Stage 0	Stage I	Stage II	Stage III	
Examination I (Preliminary)	Stage I	7 (14%)	1 (2%)	0 (0%)	0 (0%)	0.0002
	Stage II	14 (28%)	2 (4%)	9 (18%)	2 (4%)	
	Stage III	3 (6%)	1 (2%)	3 (6%)	8 (16%)	
*po* (N = 13)		Stage 0	Stage I	Stage II	Stage III	*p*
Examination I (Preliminary)	Stage I	0 (0%)	0 (0%)	0 (0%)	0 (0%)	0.2271
	Stage II	4 (31%)	1 (7.5%)	3 (23%)	0 (0%)	
	Stage III	1 (7.5%)	1 (7.5%)	1 (7.5%)	2 (16%)	

**Table 6:** The treatment efficacy based on comparing the patient’s condition on the initial and the last examination, performed after 11–12 months of PENTO therapy.

	Total	
	N	%
Complete remission	29	46
No change	23	37
Improvement	9	14
Deterioration	2	3

**Table 7:** The treatment efficacy estimated based on comparing the patient’s condition on the initial and the last examination, performed after 11–12 months of PENTO therapy, regarding the route of antiresorptive drug administration, gender, and the lesions’ location.

	Route of Bisphosphonate Administration				*p*	Sex				*p*	Location				*p*
	*iv* (N = 50)		*po* (N = 13)			F (N = 27)		M (N = 36)			Maxilla (N = 16)		Mandible (N = 47)		
	N	%	N	%	0.6532	N	%	N	%	0.9032	N	%	N	%	0.1728
Complete remission	24	48	5	38.5		11	40.5	18	50		11	68.5	18	38	
No change	18	36	5	38.5		11	40.5	12	33		3	18.5	20	43	
Improvement	6	12	3	23		4	15	5	14		2	13	7	15	
Deterioration	2	4	0	0		1	4	1	3		0	0	2	4	

**Table 8:** The treatment efficacy estimated based on comparing the patient’s condition on the initial and the last examination, performed after 11–12 months of PENTO therapy, regarding the lesions’ location.

	Quadrant 1 (N = 7)		Quadrant 2 (N = 9)		Quadrant 3 (N = 19)		Quadrant 4 (N = 28)		*p*
	N	%	N	%	N	%	N	%	
Complete remission	6	86	5	56	9	47.5	9	32	0.2216
No change	1	14	2	22	9	47.5	11	39.5	
Improvement	0	0	2	22	1	5	6	21.5	
Deterioration	0	0	0	0	0	0	2	7	

## Data Availability

The data supporting the findings of this study are available from corresponding authors upon reasonable request.
